# Factors underlying international doctoral students’ English academic writing abilities

**DOI:** 10.1371/journal.pone.0324564

**Published:** 2025-06-04

**Authors:** Wai Mar Phyo, Marianne Nikolov, Ágnes Hódi

**Affiliations:** 1 University of Szeged, Szeged, Hungary; 2 Department of English Applied Linguistics, University of Pécs, Pécs, Hungary; 3 Department of Kindergarten Teacher Training, University of Szeged, Szeged, Hungary; University of Jeddah, SAUDI ARABIA

## Abstract

The ability to write academic texts in English requires both knowledge of academic writing conventions and expertise in academic discourse to address the needs and expectations of the target academic audience in respective disciplines (Hyland, 2018). This study investigates the relationship between non-native English-speaking doctoral students’ English academic writing abilities and various factors, including their English literacy background, research knowledge, ability to read and critically evaluate academic texts, coping with emotional challenges such as stress and anxiety, as well as feedback and motivation. A total of 255 international doctoral students, speaking 49 different first languages, participated in the study. Data were collected via a survey with items presented on a 1–6 Likert scale following Dörnyei and Dewaele (2022). The analysis revealed the significant impact of English literacy background, initial academic writing abilities, and research knowledge on students’ self-assessment of academic writing skills and research competence at the outset of their doctoral studies. While prior experience in academic writing proved beneficial, it did not always translate into significant improvements in current writing abilities or research knowledge at later stages. Feedback from advisors and doctoral research course instructors emerged as vital components in supporting academic reading and motivation throughout their doctoral journey. However, the quality of peer feedback appeared to have less impact on students’ academic performance and psychological state. Overall, these findings underscore the complexity of factors influencing academic writing and research competence among NNES doctoral students, suggesting the importance of tailored support and interventions to enhance their scholarly development.

## 1. Introduction

Students who pursue PhD education often have responsibilities in their families, workplaces, and communities. Consequently, working towards the successful completion of a PhD dissertation and meeting publication requirements to graduate on time often pose challenges [1–4]. English academic writing (EAW) requires a high level of knowledge in disciplinary academic conventions as well as an understanding of the needs of the target audience (Hyland, 2018). The demands of EAW add an extra layer when novice writers do not have enough academic writing experience [3,5–9]. As PhD education is research-based, research knowledge is essential in writing up doctoral research texts [3,10–12].The ability to critically engage with academic sources also plays an integral role in PhD education [13–16]. Doctoral writing demands tend to negatively impact emotional well-being as it causes stress and anxiety [17,18]. However, the quality of students’ texts can be largely improved when they receive feedback [19,20].

**Fig 1:**
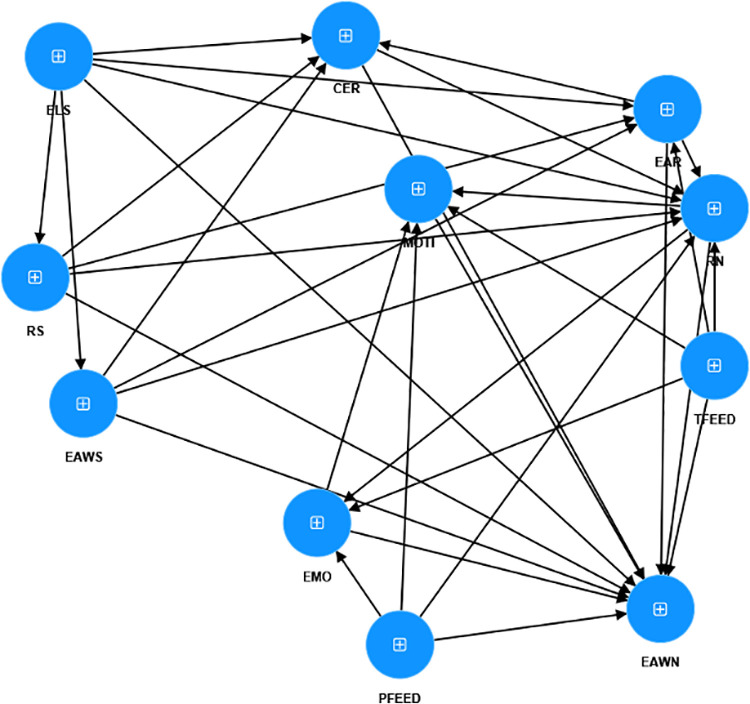
The proposed relationships among variables.

Despite substantial research on academic writing in doctoral education [21–24], there is a gap in studies on how non-native English-speaking (NNES) doctoral students’ English academic writing (EAW) abilities are influenced by their English literacy background, EAW abilities at the beginning of their PhD studies, research knowledge at the start and at the current point in PhD studies, coping with emotions, feedback, and motivation. This study aims to bridge this gap by formulating the following hypotheses (see Fig 1). Furthermore, no similar study has been conducted in the context of PhD education in Hungary where English serves as a lingua franca for both faculty and students. Therefore, the current study is innovative in this regard. In addition, the current study aims to complement the findings of previous research endeavors. This includes a study that explored the EAW support needed by NNES doctoral students (Author et al., 2024), a metaphorical study that examined how NNES doctoral students conceptualized their EAW experiences (Author et al., 2023), and studies that investigated NNES doctoral students’ self-assessment of their writing abilities (Author et al., 2023a), research knowledge and abilities (Author et al., 2022a), as well as their ability to cope with stress and anxiety (Author et al., 2022b).

### Hypotheses

1. English literacy at the start of PhD studies (ELS) positively impacts English academic writing at the start (EAWS) and at the current point/now in PhD studies (EAWN), Research knowledge at the start (RS) and at the current point/now in PhD studies (RN), English academic reading (EAR), and ability to critically evaluate reading materials written in English (CER)
2. EAWS positively impacts EAWN, RN, CER and EAR.
3. RS positively impacts CER, EAR, EAWN, RN
4. CER positively impacts EAR, EAWN, and RN.
5. EAR positively impacts EAWN, RN, ability to cope with emotions (EMO) and motivation (MOTI).
6. RN positively impacts EAWN, EMO and MOTI.
7. Teachers’ feedback (TFEED) positively impacts ENOW, RN, EMO and MOTI.
8. Peer feedback (PFEED) positively impacts ENOW, RN, EMO and MOTI.
9. EMO positively impacts MOTI and EAWN.
10. MOTI positively impacts EAW.

## 2. Method

### 2.1. Research design

This study is part of a larger research project that used mixed methods following the guidelines of Creswell (2018). This paper focuses on the quantitative phase.

### 2.2. Participants

A total of 255 international doctoral students with 48 first languages participated in the study. They were enrolled in 65 PhD programs across Hungary during the 2021–2022 academic year. They pursued their doctoral studies in a variety of research areas: agricultural science (10.6%), computer science and information technology (5.1%), economic science (8.6%), educational science (24.3%), engineering science (14.9%), medical and health science (7.5%), natural science (9.4%), and humanities (19.6%). As for their English proficiency, 8.6% had a certificate at C2, 46.3% at C1, and 45.1% at B2 level.

### 2.3. Instrument

In doctoral-level academic writing, proficiency in English literacy plays a pivotal role in effectively conveying complex ideas [3,5–8]. Therefore, to investigate participants’ evaluations of their English literacy at the start of their PhD studies (ELS), their evaluations of English academic writing at the start (EAWS) and current points/now (EAWN) of their PhD studies, three variables were included: ELS (2 items), EAWS (17 items), and EAWN (22 items). Since doctoral dissertations disseminate research works, a high level of research area knowledge and the ability to conduct doctoral research tasks are essential for successful PhD completion [3,10–12]. To assess how participants evaluated their research knowledge at the start (RS) and currently/now (RN) in their PhD studies, two variables were added: RS (7 items) and RN (7 items). PhD students are expected to work independently, making the ability to critically consult academic texts in English crucial for writing doctoral texts [13–16]. Therefore, English academic reading (EAR: 3 items) and critically evaluating reading materials (CER: 2 items) were included in the survey. The demands of doctoral writing often pose emotional challenges such as stress and anxiety [17,18]. To investigate participants’ ability to cope with these emotions, the variable coping with emotions (EMO: 2 items) was included. Quality feedback from teachers and peers can help novice writers improve the quality of their academic texts [19,20]. Hence, two variables on feedback were included: Teachers’ feedback (TFEED) and peer feedback (PFEED). Remaining motivated until doctoral completion is essential for successful PhD completion. Therefore, a variable on factors positively motivating students’ motivation (MOTI) was included. According to Ryan and Deci [25,26], learners stay motivated along their academic journey when they feel a sense of competence, autonomy, and support from their respective communities. Thus, the items in the motivation construct covered these three aspects: writing autonomy (MOTI1), writing competence (MOTI2), and the support provided by respective doctoral schools (MOTI3–5). All survey items were presented on a 1–6 Likert scale following Dörnyei and Dewaele (2022).

#### 2.3.1. Reliability and validity of the instrument.

**Fig 2:**
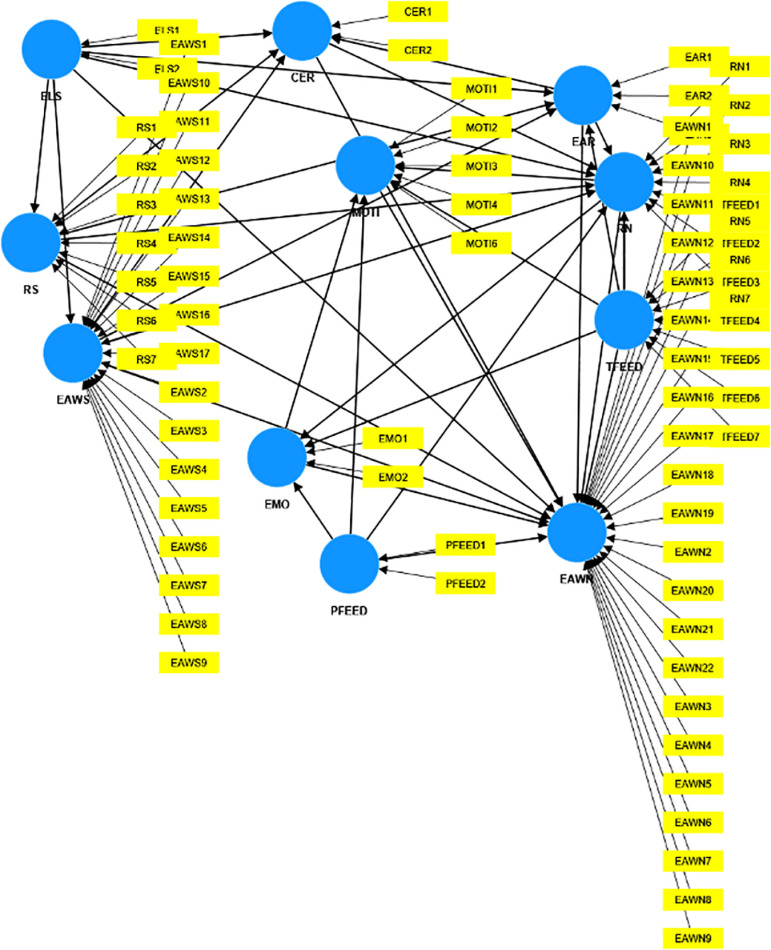
Full measurement of the outer model.

To assess the measurement of the outer model (Fig 2), an examination of factor loadings was conducted first. As a general guideline, a loading below 0.5 should be removed as they do not contribute to the measurement variable [27,28]. The construct (ELS) was originally consisted of 3 items; however, an item with a low loading of.39, therefore, this item was removed from the analysis. In the construct Motivation (MOTI), four items were excluded due to insufficient loadings falling below 0.5. All remaining items in each construct and their corresponding loadings are presented in Table 1 for comprehensive reference.

**Table 1:** Factor loadings of the items.

Code	Name of the variables and their items	Loadings
**English literacy at the start of PhD studies (ELS)**		
ELS1	My general English proficiency was at advanced level.	0.923
ELS2	I could comprehend English academic texts well.	0.923
**English academic writing abilities at the start of PhD studies (EAWS)**		
EAWS1	My special English vocabulary was not good enough to write my course assignments.	0.651
EAWS2	I knew how to write a literature review in English.	0.758
EAWS3	I did not know how to write a research paper in English.	0.740
EAWS4	I was familiar with guidelines like APA or MLA.	0.651
EAWS5	I had no experience in English academic writing.	0.763
EAWS6	I could write so that my audience understood the meaning clearly.	0.664
	At the start of PhD studies, I had a good knowledge of	
EAWS7	paraphrasing texts	0.776
EAWS8	citing and referencing sources	0.698
EAWS9	organizing paragraphs	0.845
EAWS10	grammar	0.794
EAWS11	special vocabulary	0.790
EAWS12	writing paragraphs	0.841
EAWS13	presenting ideas logically	0.881
EAWS14	stating problems clearly	0.864
EAWS15	summarizing key points	0.848
EAWS16	drawing conclusions	0.826
EAWS17	being critical	0.766
**Research knowledge at the start of their PhD studies (RS)**		
	At the start of PhD studies, I had a good knowledge of	
RS1	my research area	0.698
RS2	research design and research methodology	0.891
RS3	finding and analyzing the special literature	0.840
RS4	designing research instruments	0.882
RS5	formulating research questions	0.876
RS6	analyzing data	0.832
RS7	how to write a publishable paper in English	0.790
**English academic reading (EAR)**		
EAR1	I rarely have difficulty with comprehending technical words or phrases.	0.564
EAR2	I can understand the details in long complex texts without using a dictionary.	0.895
EAR3	I can understand journal articles without rereading difficult sections.	0.896
**Ability to critically evaluate reading materials (CER)**		
CER1	I can use my critical thinking to determine how well a publication is researched.	0.962
CER2	I can use my critical thinking to decide the validity of arguments in a text.	0.986
**English academic writing abilities at the current point in PhD studies/now (EAWN)**		
EAWN1	I can write clear, highly accurate and smoothly flowing complex academic texts.	0.560
EAWN2	I can show flexibility in formulating ideas in differing linguistic forms to convey meaning precisely.	0.750
EAWN3	I have a good command of specific vocabulary related to my larger field of study.	0.706
EAWN4	I can create coherent and cohesive texts.	0.732
EAWN5	I can use a wide range of connectors and other cohesive devices.	0.743
EAWN6	I can demonstrate consistent and highly accurate grammatical control of complex language forms.	0.764
EAWN7	Errors are rare in my texts.	0.855
EAWN8	I can write clear, smoothly flowing, complex texts.	0.868
EAWN9	I can write a critical overview of the relevant literature.	0.842
EAWN10	I can write a publishable paper on an empirical study I designed and implemented.	0.884
	At the current point in my PhD studies, I have a good knowledge of	
EAWN11	paraphrasing texts	0.846
EAWN12	citing and referencing sources	0.871
EAWN13	organizing paragraphs	0.780
EAWN14	grammar	0.852
EAWN15	special vocabulary	0.806
EAWN16	writing paragraphs	0.828
EAWN17	presenting ideas logically	0.852
EAWN18	stating problems clearly	0.760
EAWN19	summarizing key points	0.857
EAWN20	drawing conclusions	0.784
EAWN21	being critical	0.86
EAWN22	using guidelines like APA or MLA	0.874
**Research knowledge at the current point in PhD studies/now (RN)**		
	At the start of my PhD studies,	
RN1	my research area	0.863
RN2	research design and research methodology	0.850
RN3	finding and analyzing the special literature	0.899
RN4	designing research instruments	0.900
RN5	formulating research questions	0.909
RN6	analyzing data	0.881
RN7	how to write my dissertation in English	0.845
**Teachers’ feedback (TFEED)**		
TFEED1	I had access to clear guidance on the requirements for English academic writing.	0.841
TFEED2	I got helpful feedback from my tutors on the content of my written assignments.	0.88
TFEED3	I got helpful feedback from my tutors on the academic English of on my written assignments.	0.791
TFEED4	The feedback from my thesis advisor was helpful on the content of my work.	0.835
TFEED5	My thesis advisor gave me detailed feedback on the academic English of my work.	0.693
TFEED6	Tutors in my doctoral program offer consistent feedback on my English skills.	0.694
TFEED7	I could use the feedback I received to improve my written work.	0.669
**Peer feedback (PFEED)**		
PFEED8	The feedback I got from my peers helped me edit the English of my written work.	0.962
PFEED9	Peer feedback was useful about the content of my written work.	0.962
**Coping with emotions (EMO)**		
EMO1	I can handle my stress and anxiety successfully.	0.908
EMO2	I’ve managed to maintain my motivation to complete my doctoral work.	0.908
**Motivation (MOTI)**		
MOTI1	I can manage to overcome the challenges I faced in English academic writing.	0.714
MOTI2	I feel that my academic English writing abilities have improved.	0.718
MOTI3	My doctoral program offers good support in English academic writing.	0.717
MOTI4	My doctoral program has clear criteria on how written assignments in English are assessed.	0.770
MOTI5	I get all the help I need to be successful.	0.755

The reliability of the instrument was assessed following Cronbach (1951) and Hair et al. (2017): α ≥ 0.9 (excellent), 0.9 ≤ α ≥ 0.8 (good), 0.8 ≤ α ≥ 0.7 (acceptable), 0.7 ≤ α ≥ 0.6 (questionable), 0.6 ≤ α ≥ 0.5 (poor), and α ≤ 0.5 (unacceptable). The constructs in the study showed a high level of reliability and the analysis results of α value were presented in the Table 1 in Appendix 1.

The convergent validity of the constructs was assessed using Average Variance Extracted (AVE) and Composite Reliability (CR) measures (both rho_a and rho_c). AVE values should exceed 0.5 for each composite construct, while the acceptable range for CR is typically between 0.70 and 0.80, with values above 0.80 considered good, and values above 0.90 considered excellent [28,29,30]. The analysis results shown in Table 2 revealed that the underlying constructs and their relationships, providing confidence in the convergent validity of the research instrument (see Table 1 in Appendix 1).

**Table 2:** The summary of hypotheses testing results.

	Hypothesis (H)	Original sample (O)	Sample mean (M)	Standard deviation (STDEV)	T statistics (|O/STDEV|)	P values	Result
**Impact of English literacy at the start of PhD studies (ELS)**							
H1	ELS - > EAWS	−0.608	−0.612	0.037	16.425	0.000	Supported
H2	ELS - > RS	0.331	0.332	0.063	5.223	0.000	Supported
H3	ELS - > CER	0.193	0.191	0.076	2.548	0.011	Supported
H4	ELS - > EAR	0.233	0.232	0.071	3.276	0.001	Supported
H5	ELS - > EAWN	0.064	0.062	0.041	1.57	0.117	Not supported
H6	ELS - > RN	0.104	0.101	0.06	1.735	0.083	Not supported
**Impact of English academic writing abilities at the start of PhD studies (EAWS)**							
7	EAWS - > CER	−0.219	−0.222	0.079	2.768	0.006	Supported
8	EAWS - > EAR	−0.106	−0.106	0.077	1.381	0.167	Not supported
9	EAWS - > EAWN	−0.221	−0.22	0.041	5.408	0.000	Supported
10	EAWS - > RN	−0.06	−0.059	0.06	0.998	0.318	Not supported
**Impact of research knowledge at the start of PhD studies (RS)**							
11	RS - > CER	0.249	0.251	0.069	3.61	0.000	Supported
12	RS - > EAR	−0.034	−0.028	0.061	0.554	0.580	Not supported
13	RS - > EAWN	−0.121	−0.121	0.037	3.241	0.001	Supported
14	RS - > RN	0.22	0.219	0.062	3.532	0.000	Supported
**Impact of ability to critically evaluate reading materials written in English (CER)**							
15	CER - > EAWN	0.177	0.178	0.049	3.579	0.000	Supported
16	CER - > RN	0.355	0.353	0.058	6.091	0.000	Supported
**Impact of English academic reading (EAR)**							
17	EAR - > EAWN	0.189	0.191	0.047	4.004	0.000	Supported
18	EAR - > RN	0.2	0.202	0.068	2.952	0.003	Supported
19	EAR - > CER	0.515	0.513	0.065	7.88	0.000	Supported
**Impact of research knowledge at the current point in PhD studies/now (RN)**							
20	RN - > EAWN	0.474	0.471	0.066	7.138	0.000	Supported
21	RN - > EMO	0.42	0.42	0.084	4.985	0.000	Supported
22	RN - > MOTI	0.163	0.166	0.068	2.383	0.017	Supported
**Impact of teachers’ feedback (TFEED)**							
23	TFEED - > EAR	0.116	0.115	0.057	2.024	0.043	Supported
24	TFEED - > EMO	0.225	0.223	0.083	2.694	0.007	Supported
25	TFEED - > MOTI	0.534	0.535	0.053	10.144	0.000	Supported
26	TFEED - > RN	0.165	0.169	0.064	2.593	0.010	Supported
27	TFEED - > EAWN	−0.074	−0.071	0.047	1.565	0.118	Not supported
**Impact of peer feedback (PFEED)**							
28	PFEED - > EMO	0.141	0.142	0.075	1.892	0.059	Not supported
29	PFEED - > MOTI	0.012	0.012	0.051	0.244	0.807	Not supported
30	PFEED - > RN	−0.022	−0.024	0.05	0.44	0.660	Not supported
31	PFEED - > EAWN	−0.046	−0.045	0.03	1.535	0.125	Not supported
**Impact of ability to cope with emotions (EMO) and motivation (MOTI)**							
32	EMO - > MOTI	0.308	0.307	0.048	6.411	0.000	Supported
33	EMO - > EAWN	0.028	0.029	0.035	0.802	0.422	Not supported
34	MOTI - > EAWN	0.15	0.148	0.054	2.787	0.005	Supported

The discriminant validity of the instrument is also assessed using the Heterotrait-Monotrait (HTMT) and Fornell-Larcker criterion. Discriminant validity is achieved if the HTMT ratios are less than a certain threshold, often 0.9 [27,28]. According to the Fornell-Larcker criterion, if the square root of the AVE for a construct is higher than its correlations with other constructs, discriminant validity is achieved, regardless of whether the value exceeds 0.5 [27]. The analysis results revealed that the measurement model in this study achieved discriminant validity both in HTMT ratio analyses (see Table 2 in Appendix 1) and Fornell-Larcker criterion (see Table 3 in Appendix 1).

The discriminant validity of the instrument is also assessed using the Heterotrait-Monotrait (HTMT) and Fornell-Larcker criterion. Discriminant validity is achieved if the HTMT ratios are less than a certain threshold, often 0.9 [27,28]. According to the Fornell-Larcker criterion, if the square root of the AVE for a construct is higher than its correlations with other constructs, discriminant validity is achieved, regardless of whether the value exceeds 0.5 [27]. The analysis results revealed that the measurement model in this study achieved discriminant validity both in HTMT ratio analyses (see Table 2 in Appendix 1) and Fornell-Larcker criterion (see Table 3 in Appendix 1).

### 2.4. Procedure

After receiving ethical approval (see Appendix 2), the survey link, created with Google Form, was posted on PhD students’ forums. Additionally, the researcher contacted multiple directors and student representatives of doctoral institutions across Hungary, along with the survey link. The survey distribution period was from February 21, 2022, to December 30, 2022.

### 2.5. Data analysis

The data analysis was conducted utilizing Partial Least Squares Structural Equation Modeling (PLS-SEM) to assess the relationships proposed in the hypotheses. PLS-SEM is a versatile statistical technique suited for hypothesis testing, allowing for the simultaneous evaluation of measurement and structural models [27,31]. The detailed results of data analysis were presented in the Result section.

### 2.6. Ethical approval certificate from the Institutional Review Board (IRB)

The very first step in the research process was applying for ethical approval. The detailed research plan was submitted to the Institutional Review Board (IRB) of the Doctoral School of Education at the University of Szeged, along with the research timeline. After a thorough review process, the application received approval. The application provided a thorough overview of the research strategy, clearly outlining essential elements such as voluntary participation, the use of anonymized coding, the adoption of inclusive language in reporting research results, the dedication to using data exclusively for research aims, and the plan to share research findings in international research settings and relevant academic communities. After a meticulous assessment by the IRB, the research endeavor obtained ethical clearance (ref #: 17/2021; refer to Appendix 2). The survey was then distributed to international doctoral research institutions. Participants in the study were themselves doctoral researchers (therefore, there was no minor). and well-informed about research practices. The survey message also explicitly stated that participation was entirely voluntary, and participants were assured that their data would be coded and used exclusively for informing stakeholders about the challenges and requirements in doctoral-level academic writing faced by doctoral students during the fulfillment of their doctoral requirements. The researcher’s contact information, including affiliation and email address, was made available to facilitate participant communication at any time. The students provided informed consent to take part in the study and the majority of participants provided their email addresses, volunteering to participate in follow-up interviews.

## 3. Results

### 3.1. Assessment of the structural model

**Fig 3:**
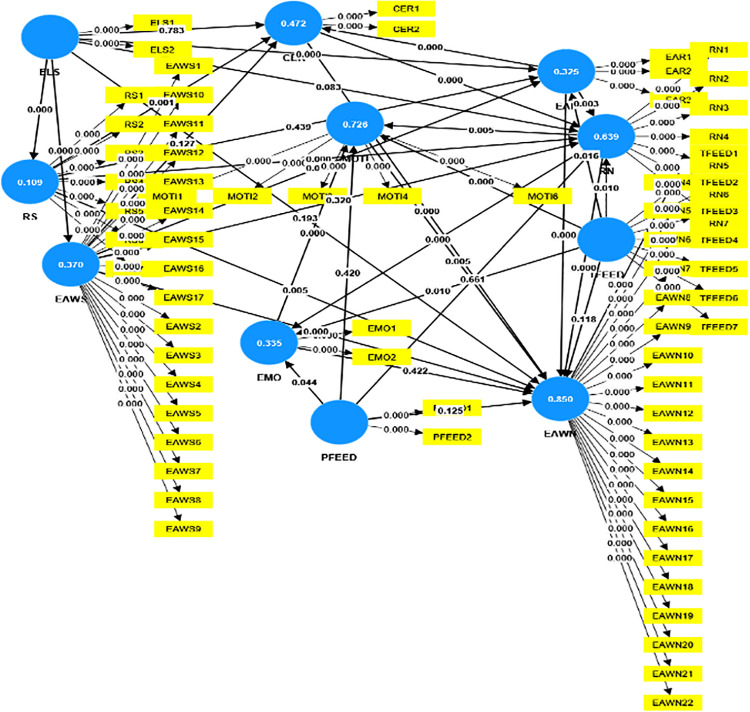
The structural model of the proposed model.

In assessing structural model (Fig 3), multicollinearity between the predictors were evaluated first as high levels of collinearity can affect the stability of estimates. In structural equation modeling, Variance Inflation Factor (VIF) values less than 5 are considered acceptable to indicate low multicollinearity: VIF < 5 (acceptable), 5 < VIF < 10 (moderate), VIF > 10 (unacceptable). In this study, the VIF values of all the latent variables were less than 5 (see Table 4 in Appendix 1), indicating no collinearity issues [27,32].

As the second step, the structural model’s explanatory power was evaluated through R² values following [27,28]. According to the results, approximately 28.9% of the variability in CER was accounted for by the predictor variables in the model, suggesting that the identified factors contributed moderately to explaining the observed variability in CER. EAR demonstrated a higher explanatory capability, with 49% of its variance explained. EAWN exhibited a substantial 84.1% variance explained, indicating that the selected factors strongly contributed to explaining the observed variability EAWN. EAWS and EMO were explained by their predictors at 36.3% and 34.2%, respectively. MOTI showed a strong explanatory power with 73.3% variance explained. RN was characterized by 63.9% variance explained, while RS had a lower explanatory power, with 10.9% of its variance explained.

In the third step, effect sizes between constructs were analyzed using f^2^ values; f^2^ values around 0.02 indicate small effects, 0.15 medium effects, and 0.35 large effects [27,31]. ELS showed a large effect size on EAWS (f^2^ = 0.586) and a medium size on RN (f^2^ = 0.123). EAWS had a medium effect on EAWN (f^2^ = 0.146), and a small effect on EAR (f^2^ = 0.037). RS displayed a small effect on CER (f^2^ = 0.053), RN (f2 = 0.043), and EAWN (f^2^ = 0.084). CER had medium effects on EAWN (f2 = 0.102) and a medium effect on RN (f2 = 0.181). EAR abilities showed CER (f^2^ = 0.349) and a medium effect on EAWN (f^2^ = 0.115). RN had a large effect on EAWN (f^2^ = 0.454) and a medium effect on EMO (f^2^ = 0.174). TFEED resulted in a large effect on MOTI (f^2^ = 0.585) and small effect size on EAR, EAWN, EMO and RN. Peer feedback (PFEED) showed small effects on EAR, EAWN, EMO and RN all constructs (f^2^ = 0.004 to 0.022).

As the next step, the Q² values were assessed to evaluate the predictive relevance of the model. Q² values above 0.25 indicate small effects, around 0.50 moderate effects, and beyond 0.75 large effects [27,32].

The results revealed that the model had small predictive relevance for CER with a Q² of 0.182. For EAR, the model demonstrated medium predictive relevance, as indicated by a Q² of 0.269. Moreover, the model exhibited high predictive relevance for EAWN, with a Q² value of 0.377. Similarly, the model showed strong predictive capabilities for EAWS, as reflected by a Q² of 0.352. In terms of EMO, the model indicated moderate predictive relevance with a Q² of 0.211. MOTI emerged as a strongly predictable construct, with a Q² of 0.607, suggesting that the identified factors contribute significantly to forecasting motivation. RN shows moderate predictive relevance with a Q² of 0.307, while RS had limited but relevant predictive capabilities, indicated by a Q² of 0.097.

Finally, bootstrapping analyses were conducted at a 5% significance level to ensure robustness and reliability in assessing the structural model’s parameters, significance levels, and to test hypotheses. All the results of hypotheses testing were summarized in Table 2 and in Fig 3.

## 4. Discussion

The discussion will focus on the hypotheses testing results (Table 2) and their significance for understanding the dynamics of English academic writing developmental process.

The results (*H1-6*) revealed that students’ English literacy at the start of their PhD studies (ELS) had a significant impact on their self-assessment of English academic writing abilities and at the start of their PhD studies (EAWS, p < .01), supporting the hypothesis that general English literacy helped NNES students to write academic texts in English. Moreover, ELS’s significant impact on research knowledge at the start of the PhD studies (RS, p < .001) revealed that English proficiency helped them in developing their research knowledge. ELS’s significant impact on CER (p < .05) and EAR (p < .05) showed that English literacy background helped the NNES students in this study when they critically evaluated reading academic materials that were written in English, and it also enhanced their reading proficiency as well.

However, the results did not provide enough statistical evidence to prove (p > .05) that it had a significant impact on English academic writing (EAWN) and research knowledge (RN) at the current point in PhD studies, even though it had a positive relationship with both EAWN and RN. Therefore, the results indicated that English proficiency is important in developing academic writing abilities in English language, as scholars have pointed out [5,8]. Moreover, the detailed statistical result of ELS’s impact on EAWS, which was shown in Table 2, suggests that the significantly positive impact was seen among students with lower English proficiency levels, rather than those with the highest proficiency levels, revealing that the highest proficiency level students were critical in assessing their writing ability at the expected standardized scholarly level using academic English. This result is consistent with the two previous studies in which the same students participated (Author et al., 2023, 2024). Author et al. (2023) revealed that statistically significant higher self-assessed scores were found among lower English proficiency level students at the p < .01 level, while C2 level students tended to show fewer significant differences. Similarly, Author et al. (2024) found that students with high English proficiency levels were critical in assessing their academic writing ability and were also able to identify the exact areas of academic writing in which they needed to improve. For example, a student at C2 English proficiency level wanted structured writing instruction tailored to meet the demands of academic writing in English because they needed to improve their writing skill in “formulating the argument and retaining a coherent balance of the argument throughout the paper” and to familiarize themselves “more with different stylesheets to keep the integrity of the article intact” (Author et al., 2024, p. 3). In line with previous research by the authors, this study also revealed that students with the highest English proficiency were critical in assessing their ability to write academic text at an expected scholarly level in English. Overall, in this study context, a significantly positive impact of English literacy was found among the majority of the participants, not among C2 level students which were just a minority as only 8.6% of the participants were at the C2 level in this research context.

In terms of the results (*H7-10*) about the impact of English academic writing at the start of PhD studies (EAWS), EAWS showed a significantly positive impact on the students’ ability to critically evaluate reading materials (CER, p < .01) and their current writing abilities (EAWN, p < .001), indicating that their previous experience in academic writing helped them when they critically consulted existing literature and when they dealt with academic writing tasks; however, the statistics also suggest that current higher CER and EAWN ability might not always be explained by initial writing ability, implying that even the students who were not confident about their writing abilities at the start of PhD studies were able to critically engage with the literature at the current point in their PhD studies and conduct doctoral writing tasks well (*H7, H9*). However, EAWS did not reveal enough statistical evidence to prove that it had a significant impact on current English academic reading abilities (EAR, p > .05) and current research knowledge (RN, p > .05), therefore, not supporting the hypotheses. Therefore, the current study indicated that it is better for the students of NNES background if they have prior academic writing experience to deal with doctoral-level writing [4,7,8,23, Author et al, 2023, 2024].

According to the results (*H11-14*) on the impact of (RS), research knowledge at the start of PhD studies had a statistically significantly positive impact on CER (p < .001), revealing that a good foundation in research helped the students to critically evaluate research papers written in English. In terms of RS’s impact on EAR (p > .05), the result did not support the hypothesis, indicating that these participants were developing their English academic reading skill even if their prior research knowledge upon PhD entry was not very high. The results revealed that prior research knowledge also helped the students when they wrote academic papers along their PhD journey (EAWN, p < .01). Moreover, the result clearly indicated at the p < .001 level that prior research knowledge is an essential factor in developing research knowledge at the doctoral level (RN, p < .001), supporting the hypothesis, in line with literature [11,12,35, Author et al., 2022].

In all academic papers at the PhD education level, students are required to critically approach disseminating their findings in textual forms as well as in conducting research tasks [15,16]. In this study, the positive and significant impact of CER on EAWN (p < .001) and (RN, p < .001) revealed that the ability to critically evaluate the reading materials helped develop their abilities in both academic writing (*H15*) and research knowledge (*H16*).

As PhD students are expected to carry out research tasks in an independent and effective manner, reading is the main input for the novice scholars in developing their academic abilities [13,14]. The significantly positive impact of EAR on EAWN, RN, and CER at the p < .01 level revealed that the ability to read English academic texts develops their writing, research knowledge, and critical thinking skill (*H17-19*).

Doctoral texts are about disseminating research ideas [3,10] and in this study the positive and significant impact of RN on EAWN(p < .001), EMO (p < .001), MOTI (p < .017) revealed that current research knowledge significantly helped students with their writing. Moreover, their ability in research tasks also helped them in managing anxiety and stress caused by writing demands, and to stay motivated until the doctoral work was finished (*H20-22*).

Feedback played a key role in improving the quality of novice scholars’ academic abilities [19,20] and the significant positive impact of TFEED on EAR (p < .05), EMO (p < .01), MOTI (p < .01), and RN (p < .05) revealed that teachers’ feedback helped students read more effectively as well as to cope with negative emotions caused by writing and to maintain motivation along the doctoral journey (*H23-26*). However, the statistics about TFEED impact on EAWN revealed that proficient academic writers in English were critical about the quality of feedback they received from thesis advisors and tutors (*H27*), indicating that they had a higher level of expectation.

In terms of PFEED’s (peer feedback) impact on EMO, MOTI, RN, and EAWN (*H28-31*), the statistics revealed no significant evidence (p > .05) to support the hypothesis despite the positive correlation among them, indicating that the quality of peer feedback did not contribute to participants’ writing and research abilities or their psychological well-being. The EMO’s impact on MOTI (p < .001) revealed that the ability to manage one’s emotions caused by writing anxiety could help students to remain motivated until they completed their doctoral studies (*H32*). However, EMO did not show enough statistical evidence to support the hypothesis even though EMO showed a positive correlation with students’ current writing ability (*H33*).

MOTI’s statistically significant impact on EAWN (p < .01) revealed that students maintained their motivation while meeting demands of English academic writing when they felt a sense of autonomy, competence, and being supported by their respective communities (*H34)* in line with STD theory [26,33].

## 5. Conclusion

Findings highlighted the significant impact of English literacy background, initial academic writing abilities, and research knowledge on students’ self-assessed level of their academic writing skills and research competence at the outset of their doctoral studies. The findings underpin the crucial role of critical evaluation of reading materials and the development of English academic reading skills in enhancing academic writing and research knowledge. Feedback from teachers emerged as a vital component in supporting students’ reading comprehension, emotional well-being, and motivation throughout their doctoral journey. However, peer feedback had less impact on students’ academic performance and psychological state. Overall, these findings underscore the complexity of factors influencing English academic writing among NNES doctoral students, suggesting the importance of tailored support and interventions to enhance their scholarly development.

## 6. Limitations and future directions

Limitations of this study include the reliance on self-reported data, which may introduce response bias and limit the generalizability of findings to other countries with different academic environments and policies. Future research could incorporate objective measures of academic writing proficiency, such as textual analyses of students’ writing, and expand the sample to include participants from diverse geographical backgrounds. Additionally, involving key stakeholders, such as faculty members and advisors, could enhance the robustness of the findings and provide a more comprehensive understanding of the EAW journey of NNES doctoral students. Moreover, a follow-up study using qualitative data could complement this research, offering deeper insights into the challenges and experiences of NNES doctoral students in academic writing.

## Supporting information

S1 Appendix (PDF)

S2 Appendix (PDF)
