# Supplementary material for: Factors underlying international doctoral students’ English academic writing abilities
Source: PLoS One. 2025 Jun 4;20(6):e0324564. doi: 10.1371/journal.pone.0324564 (PMC12136304; doi:10.1371/journal.pone.0324564)
Supplement: S1 Appendix — (PDF) [file pone.0324564.s001.pdf]

Table 1. Values of reliability and convergent validity

	Alpha	CR (rho_a)	CR (rho_c)	AVE
CER	0.913	0.913	0.958	0.920
EAR	0.708	0.790	0.837	0.641
EAWN	0.979	0.981	0.981	0.701
EAWS	0.943	0.949	0.950	0.531
ELS	0.826	0.826	0.920	0.851
EMO	0.788	0.788	0.904	0.825
MOTI	0.791	0.795	0.857	0.546
PFEED	0.920	0.923	0.962	0.926
RN	0.949	0.949	0.958	0.766
RS	0.925	0.926	0.940	0.692
TFEED	0.882	0.887	0.909	0.592

Table 2. Discriminant validity based on HTMT

	CER	EAR	EAWN	EAWS	ELS	EMO	MOTI	PFEED	RN	RS	TFEED
CER											
EAR	0.778										
EAWN	0.782	0.830									
EAWS	0.495	0.547	0.669								
ELS	0.469	0.667	0.648	0.686							
EMO	0.364	0.327	0.515	0.389	0.268						
MOTI	0.406	0.408	0.556	0.307	0.362	0.819					
PFEED	0.202	0.096	0.143	0.099	0.041	0.417	0.591				
RN	0.745	0.733	0.879	0.56	0.546	0.572	0.613	0.233			
RS	0.467	0.379	0.479	0.584	0.379	0.46	0.427	0.272	0.592		
TFEED	0.33	0.303	0.358	0.219	0.181	0.543	0.844	0.658	0.447	0.373	

Table 3. Discriminant validity based on Fonel Locker criterion

	CER	EAR	EAWN	EAWS	ELS	EMO	MOTI	PFEED	RN	RS	TFEED
CER	0.959										
EAR	0.640	0.801									
EAWN	0.740	0.725	0.837								
EAWS	-0.469	-0.478	-0.651	0.729							
ELS	0.408	0.502	0.582	-0.608	0.923						
EMO	0.309	0.255	0.455	-0.341	0.216	0.908					
MOTI	0.340	0.309	0.485	-0.262	0.287	0.651	0.739				
PFEED	0.185	0.078	0.135	-0.061	0.035	0.356	0.506	0.962			
RN	0.694	0.621	0.849	-0.534	0.484	0.494	0.527	0.219	0.875		
RS	0.430	0.328	0.457	-0.535	0.331	0.394	0.367	0.253	0.556	0.832	
TFEED	0.304	0.260	0.342	-0.198	0.158	0.456	0.778	0.585	0.416	0.343	0.769

Table 4. Collinearity VIF values assessment

	**CER**	**EAR**	**EAWN**	**EAWS**	**ELS**	**EMO**	**MOTI**	**PFEED**	**RN**	**RS**	**TFEED**
CER		1.455	2.282						1.931		
EAR			2.092						1.981		
EAWN						3.615	3.656				
EAWS	1.923	1.996	2.042						2.034		
ELS	1.57	1.623	1.764	1.000					1.732	1.000	
EMO							1.521				
MOTI											
PFEED			1.568			1.537	1.576		1.567		
RN			2.767			3.836	3.918				
RS	1.376	1.559	1.718						1.581		
TFEED		1.177	1.771			1.754	1.821		1.695		
